# Programmed Cell Death Ligand 1 Expression on Immune Cells and Survival in Patients With Nonmetastatic Head and Neck Cancer: A Systematic Review and Meta-analysis

**DOI:** 10.1001/jamanetworkopen.2023.6324

**Published:** 2023-03-31

**Authors:** Tomáš Blažek, Marek Petráš, Lukáš Knybel, Jakub Cvek, Renata Soumarová

**Affiliations:** 1 Department of Oncology, Ostrava University Hospital, Ostrava, Czech Republic; 2 Faculty of Medicine, University of Ostrava, Ostrava, Czech Republic; 3 Department of Epidemiology and Biostatistics, Third Faculty of Medicine, Charles University, Prague, Czech Republic; 4 Department of Oncology, Královské Vinohrady University Hospital, Prague, Czech Republic

## Abstract

**Importance:**

The failure or success of radical treatment in patients with head and neck squamous cell carcinoma (HNSCC) is associated with many known and unknown factors; hence, there is a search for further prognostic markers to help optimize therapeutic strategy and improve treatment outcomes.

**Objective:**

To assess the association of programmed cell death ligand 1 (PD-L1) expression on immune or tumor cells, including its composite expression on both cell types, with overall survival (OS) or specific survival.

**Data Sources:**

MEDLINE, Embase, PQSciTech, and HCAPlus databases were systematically searched for cohort studies focused on the prognostic role of PD-L1 expression in patients with HNSCC in curative stages of the disease. Search results generated publications from January 1, 2010, to January 6, 2023.

**Study Selection:**

Of 3825 publications identified, a total of 17 cohort studies in the English language met inclusion criteria of this systematic review and meta-analysis. Eligible studies reported adjusted hazard ratios (aHRs) with 95% CIs for the association of PD-L1 expression levels with OS and arbitrary specific survival.

**Data Extraction and Synthesis:**

Data from studies were extracted independently by 2 researchers strictly adhering to the Preferred Reporting Items for Systematic Reviews and Meta-analyses reporting guidelines and recommendations. The risk of bias was assessed using the Quality in Prognosis Studies tool and Newcastle-Ottawa Scale. Pooled effect estimates were obtained using a random-effect or fixed-effect model based on homogeneity of studies.

**Main Outcomes and Measures:**

The primary outcome was to investigate whether there was an association between PD-L1 expression on immune or tumor cells and OS.

**Results:**

In 17 cohort studies of the association of PD-L1 expression with survival in 3190 patients with HNSCC, high PD-L1 expression on immune cells was associated with a favorable OS (pooled aHR, 0.39; 95% CI, 0.25-0.59). There was no association between composite PD-L1 expression on immune and tumor cells and OS (pooled aHR, 0.79; 95% CI, 0.55-1.14) or between PD-L1 expressed only on tumor cells and OS (pooled aHR, 1.22; 95% CI, 0.87-1.70). A high level of PD-L1 expression on immune cells was associated with favorable specific survival (pooled aHR, 0.52; 95% CI, 0.38-0.72). There were no interactions between tumor location or type of primary treatment (ie, surgery vs radiotherapy or radiochemotherapy) and the association between PD-L1 expression and OS.

**Conclusions and Relevance:**

This study’s findings suggest that PD-L1 expression on immune cells may serve as a new prognostic biomarker in patients with HNSCC. However, future studies may be warranted to verify this potential role given the limited number of studies on this topic conducted and published to date.

## Key Points

**Question:**

Is programmed cell death ligand 1 (PD-L1) expression in the tumor microenvironment of patients with curative-stage head and neck squamous cell carcinoma (HNSCC) associated with overall and specific survival?

**Findings:**

This systematic review and meta-analysis of 17 cohort studies with 3190 participants of PD-L1 expression found that high PD-L1 expression levels exclusively on immune cells were associated with prolonged overall and specific survival in patients with localized and locoregionally advanced HNSCC, while PD-L1 expression on tumor cells was not associated with survival.

**Meaning:**

In this systematic review and meta-analysis, the strength of evidence was limited by the small number of studies, which suggests that future research should seek to investigate whether PD-L1 expression on immune cells is associated with survival in patients with HNSCC undergoing curative therapy.

## Teaser

This systematic review and meta-analysis investigates the association of programmed cell death ligand 1 expression levels on tumor and immune cells with survival in patients with nonmetastatic head and neck cancer.

## Introduction

In recent years, the concept of the tumor immune microenvironment, with the presence of tumor cells and infiltrating immune cells, has been intensively studied. Of particular interest are the complex interactions between tumor and immune cells that are involved in the dynamic balance between tumor control and progression.^1,2,3,4,5^ Several studies^6,7,8^ have reported the potential prognostic relevance of tumor-infiltrating T lymphocytes in various types of cancer, including studies conducted in patients with head and neck cancers. Specifically, head and neck squamous cell carcinoma (HNSCC) is considered a tumor with high immunogenic potential.^5,9^

An integral component of the tumor immune microenvironment is the immunosuppressive activity represented by inhibitory signaling molecules expressed on tumor and immune cells. A major molecule associated with tumor growth is programmed cell death ligand 1 (PD-L1). It suppresses the cytotoxic immune response mediated by CD8+ tumor–infiltrating T lymphocytes by stimulating programmed cell death 1 protein (PD-1) receptors localized on lymphocyte surfaces.^10^ While the mechanism of action of PD-L1 and PD-1 complex is well known, the prognostic role of PD-L1 expression in patients with HNSCC remains largely uncertain. Some studies have found a positive association between high PD-L1 expression and improved disease-specific survival, while others have reported a negative association.^11,12,13^ Therefore, we performed this systematic review and meta-analysis to investigate whether PD-L1 expression is associated with survival in patients with curative-stage HNSCC.

## Methods

This systematic review and meta-analysis was prepared in accordance with the recommendations of the Meta-analysis of Observational Studies in Epidemiology (MOOSE) reporting guideline and Preferred Reporting Items for Systematic Reviews and Meta-analyses (PRISMA) reporting guideline. The study protocol was registered in the international prospective register of systematic reviews. The Medical Literature Analysis and Retrieval System Online (MEDLINE), PubMed, Excerpta Medica Database (Embase), ProQuest Science and Technology (PQSciTech), and Chemical Abstracts Plus (HCAPlus) were used in our computerized search combined with manual search for relevant publications using keywords in titles. The search strategy aimed to identify English-language articles and scientific manuscripts published between January 1, 2010, and January 6, 2023, focused on the association of PD-L1 expression with outcomes among patients with HNSCC.

The following main keywords and their synonyms were used: PD-L1 expression coupled with head and neck cancer or head and neck squamous cell carcinoma (eTable 1 in Supplement 1). Studies were considered eligible if they met the following inclusion criteria: was a cohort study of patients with HNSCC in a curative stage of the disease (ie, localized and locoregionally advanced tumors), investigated the association between PD-L1 expression and overall survival (OS) expressed by adjusted hazard ratios (aHRs) with 95% CIs, a biomarker detected by immunohistochemical analysis, included treatment modalities of surgery and radiotherapy or radiochemotherapy, and investigated human papillomavirus (HPV)–negative oropharyngeal carcinomas. Excluded were studies investigating paranasal sinus tumors, thyroid or nasopharyngeal carcinomas, salivary gland tumors, mucosal melanoma, skin carcinoma, or lymphomas or occult primary tumors, as well as studies involving recurrent disease, previous radiotherapy, or distant metastatic disease. Likewise, studies assessing only HPV-positive carcinoma with different prognoses and biological natures were ineligible for inclusion.

### Data Extraction and Assessment of Risk of Bias

Data extraction, performed separately by 2 authors (T.B. and M.P.), included the study first author and title, year of publication, sample size, type of tissue (resected or biopsy), PD-L1 expression levels on tumor or immune cells, immunohistochemical method with membrane or cytoplasmatic staining and cutoff values for PD-L1 positivity, median follow-up time, and survival outcomes and study patient tumor site, stage, grade, HPV status, and treatment modality. Quantitative synthesis was conducted using extracted estimates (ie, aHRs and 95% CIs for OS or specific survival). Study risk of bias (RoB) was assessed using the Quality in Prognosis Studies (QUIPS) tool and Newcastle-Ottawa Quality Assessment Scale.^14,15^ Risk of bias was evaluated by 2 independent assessors (T.B. and M.P.), with discrepancies discussed and resolved by consensus.

### Study End Points

The primary end point was OS, defined as the period from initial radical treatment to death for any cause or the time of the last follow-up visit. A secondary end point, specific survival, was the composite of various non-OS outcome types used in selected studies. While tumor and nodal persistence or recurrence of carcinoma were defined by locoregional failure, any type of recurrence was assessed using progression-free survival, disease-free survival, or relapse-free survival. These measures were determined from the date of radiotherapy completion (for locoregional failure), initial treatment (for progression-free survival), or surgery or radiotherapy (for relapse-free survival) to the date of detection of any recurrence or relapse, death from any cause, or the date of last follow-up visit, respectively. Disease-specific survival was established as the time from initial treatment to death from cancer.

### Statistical Analysis

Study-reported aHRs for investigated factors were used for quantitative analysis. The association of PD-L1 expression on immune or tumor cells with OS or specific survival was estimated from pooled aHRs with 95% CIs. The decrease in risk of death was calculated as (1 − pooled aHR) × 100%.

The pooled outcome was obtained using a random-effects model (DerSimonian-Laird method)^16^ or a fixed-effects model (inverse variance method) based on homogeneity among studies. If the inconsistency index *I*^2^ was higher than 25% and the *P* value was lower than .10, then interstudy heterogeneity was considered and the pooled aHR was obtained from a random-effects model; otherwise, a fixed-effects model was used.^17^ Moreover, changes in outcomes associated with small studies and missing studies were tested as follows: changes in outcomes associated with small studies were determined by the regression model with Egger test, and the summary change in outcome associated with asymmetry while identifying unpublished studies was estimated by the trim and fill method.^18^

In addition, metaregression of investigated associations was conducted to investigate the association of covariates (ie, type of cells, tumor locality, and treatment modality) with outcomes. Statistical analyses were performed using Stata/BE statistical software version 17.0 (StataCorp). All tests were 2-tailed, with the level of significance set at .05.

## Results

**Figure 1.**
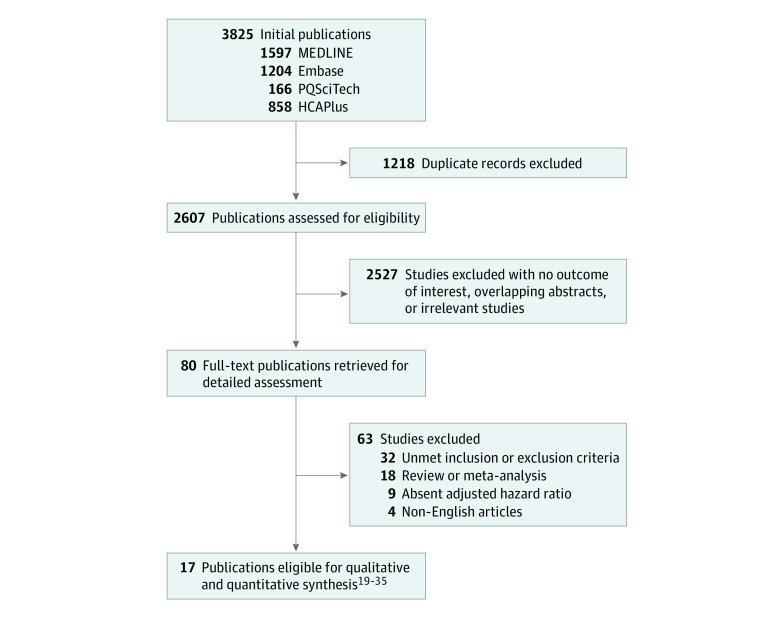
Study Flowchart

A total of 3825 publications were identified, and of 82 eligible articles, 17 studies^19,20,21,22,23,24,25,26,27,28,29,30,31,32,33,34,35^ met inclusion criteria and hence were considered eligible for our meta-analysis (Figure 1). Characteristics of these studies conducted in a total of 3190 patients with HNSCC at various sites (including the oral cavity, oropharynx, larynx, and hypopharynx) with stage I through IV disease who underwent radical curative treatment are reported in Table 1. The median (IQR) age ranged from 36 (15-45) years^27^ to 67 (50-89) years,^19^ and the proportion of patients by sex ranged from 14 female patients among 372 total patients (3.8%)^23^ to 135 female patients among 517 total patients (26.1%).^30^

**Table 1.** Characteristics of Studies

Study	No. of patients (cancer site)	Sex, No. male/No. female	Age, median (IQR), y	Treatment	Follow-up, median (IQR), mo	PD-L1 expression	Immunohistochemistry assay	Survival outcome	Determination of PD-L1 positivity	RoB^a^
Fukushima et al,^19^ 2018	92 (OP)	77/15	67 (49)	RT, CRT	36 (59)	ICs, TCs	M + C	OS, PFS	IRS	Low
Kim et al,^20^ 2016	204 (OC), 122 (OP), 44 (L), 28 (HP), 4 (other)	302/100	58 (66)	S, S + CRT	46 (NR)	ICs, TCs	M + C	OS, RFS	IRS	Low
Sato et al,^21^ 2019	137 (OP)	113/24	63 (25)	S, S + RT, CRT	37 (202)	ICs, TCs	M + C	OS, DFS	IRS	Low
Balermpas et al,^22^ 2017	41 (OC), 88 (OP), 22 (HP)	131/30	57 (NR)	S, S + CRT	48 (96)	ICs and TC	M + C	OS	IRS	Low
Sánchez-Canteli et al,^23^ 2020	24 (OP), 67 (L), 64 (HP)	358/14	59 (56)	S, S + RT	22 (NR)	ICs and TCs	M	OS, DSS	TPS, CPS	Moderate
Lilja-Fischer et al,^24^ 2020	303 (OP)	217/86	64 (NR)	RT, CRT	64 (84)	ICs and TCs	M	OS, LRF, DSS	CPS	Moderate
Ngamphaiboon et al,^25^ 2019	94 (OC), 31 (OP), 47 (L), 26 (HP), 5 (other)	145/58	64 (59)	S, S + RT, CRT	40 (NR)	ICs and TCs	M + C	OS	IRS	Low
Pena-Cardelles et al,^26^ 2022	65 (OC)	40/25	65 (NR)	S, S + CRT	73 (51)	TCs	M	OS, DFS	TPS	Low
Hanna et al,^27^ 2018	81 (OC)	49/32	36 (30)	S, S + RT, CRT	74 (226)	TCs	M + C	OS, DFS	TPS	Moderate
Hong et al,^28^ 2016	99 (OP)	79/20	58 (49)	S, S + RT, CRT	56 (183)	TCs	M	OS, LRF	TPS	Low
Zhou et al,^29^ 2020	36 (L), 38 (OC), 9 (HP), 2 (other)	67/18	57 (49)	S, S + CRT	62 (64)	TCs	M	OS, RFS	TPS	Low
Lyu et al,^30^ 2019	391 (OC), 116 (L), 10 (HP)	382/135	NR	S, S + RT, CRT	35 (211)	TCs	M	OS, RFS	TPS	Moderate
Lin et al,^31^ 2015	305 (OC)	236/69	NR	S, S + CRT	46 (132)	TCs	M + C	OS	NR	Moderate
Fu et al,^32^ 2022	63 (HNSCC)	45/18	65 (37)	S, S + CRT	72 (148)	TCs	M + C	OS, DFS	TPS	Moderate
Adamski et al,^33^ 2021	95 (OC)	63/32	NR	S, S + CRT	46 (129)	TCs	M + C	OS	TPS	Low
Yang et al,^34^ 2018	64 (HNSCC), 17 (OP)	65/16	NR	S, S + CRT	41 (96)	TCs	M + C	OS, DFS	TPS	Low
Schneider et al,^35^ 2018	36 (OC), 58 (OP), 14 (L), 21 (HP)	97/28	NR	S, S + CRT	121 (161)	TCs	M + C	OS, DFS	TPS	Moderate

Abbreviations: C, cytoplasmic staining; CPS, combined positive score; CRT, chemoradiotherapy; DFS, disease-free survival; DSS, disease-specific survival; HNSCC, head and neck squamous cell carcinoma with no specified site; HP, hypopharyngeal; IC, immune cell; IRS, immunoreactivity score; L, laryngeal; LRF, locoregional failure; M, membranous staining; NR, not reported; OC, oral cavity; OP, oropharyngeal; OS, overall survival; PD-L1, programmed cell death ligand 1; PFS, progression-free survival; RFS, relapse-free survival; RoB, risk of bias; RT, radiotherapy; S, surgery; S + CRT, surgery followed adjuvant chemoradiotherapy; S + RT, surgery followed adjuvant radiotherapy; TC, tumor cell; TPS, tumor proportion score.

^a^ Assessed with Quality in Prognosis Study tool.

In all studies, formalin-fixed, paraffin-embedded hematoxylin and eosin–stained tissue sections were used for PD-L1 detection. Specimens were obtained by surgical resection (15 studies^20,21,22,23,25,26,27,28,29,30,31,32,33,34,35^), biopsy (2 studies^19,24^), or both methods (5 studies^21,25,27,28,30^). PD-L1 expression on tumor or immune cells was measured by immunohistochemical analysis using monoclonal or polyclonal antibodies. The level of PD-L1 positivity, not reported in 1 study,^31^ was determined using the tumor proportion score in 10 studies,^23,26,27,28,29,30,32,33,34,35^ immunoreactivity score in 5 studies,^19,20,21,22,25^ and combined positive score (CPS) in 2 studies.^23,24^

### Association of PD-L1 Expression With Survival

**Figure 2.**
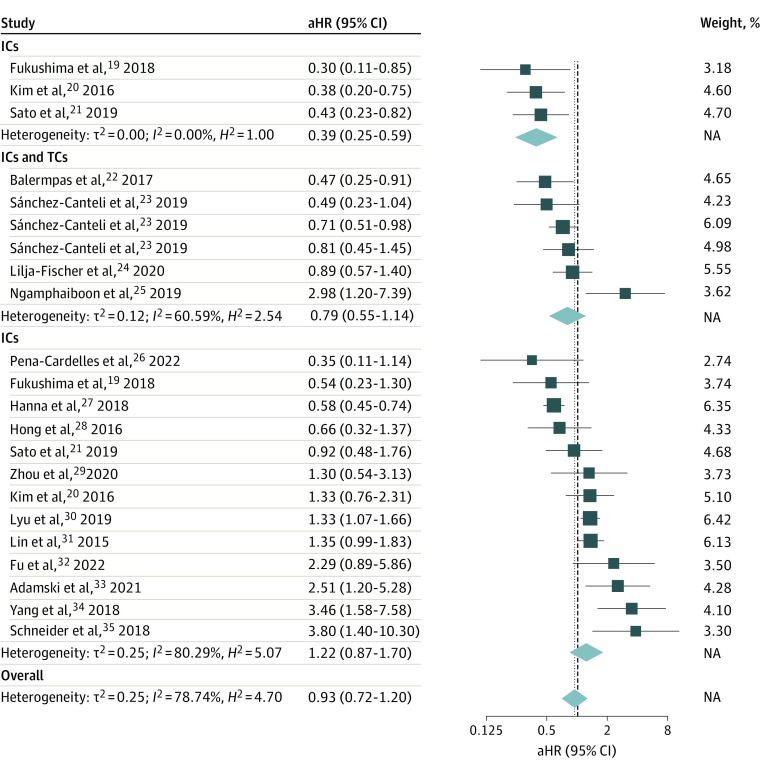
Association of Programmed Cell Death Ligand 1 Expression Levels With Overall Survival Results are presented from a random-effects model, including τ^2^ (heterogeneity variance), *I*^2^ (inconsistency index), *H*^2^ (*H* statistics), and *P* value. aHR indicates adjusted hazard ratio; box size, weight of the aHR (effect size); dashed lines, overall pooled aHRs; diamonds, pooled aHRs; IC, immune cell; lateral points, 95% CIs; NA, not applicable; TC, tumor cell.

The pooled aHR of 0.93 (95% CI, 0.72-1.20) showed no association between PD-L1 expression on tumor or immune cells and OS (Figure 2). However, different outcomes were found when PD-L1 expression was stratified by cell type. High PD-L1 expression on immune cells was associated with OS (pooled aHR, 0.39; 95% CI, 0.25-0.59). There was no association between composite PD-L1 expression on tumor or immune cells and OS (pooled aHR, 0.79; 95% CI, 0.55-1.14) or ligand expression on tumor cells only and OS (pooled aHR, 1.22; 95% CI, 0.87-1.70).

Mutually adjusted metaregression coefficients demonstrated significantly more favorable OS in patients with PD-L1 highly expressed on immune vs tumor cells (regression coefficient, −1.15; 95% CI, −1.95 to −0.35; *P* = .005). Composite PD-L1 expression on both cell types was not associated improved OS or specific survival compared with PD-L1 expressed on only tumor cells (Table 2).

**Table 2.** Outcomes of Subgroup Meta-analysis and Metaregression

Factor	Overall survival				Specific survival			
	Estimates, No.	Pooled aHR (95% CI)	Regression coefficient (95% CI)	*P* value	Estimates, No.	Pooled aHR (95% CI)	Regression coefficient (95% CI)	*P* value
PD-L1 expression location								
ICs	3	0.39 (0.25 to 0.59)	−1.15 (−1.95 to −0.35)	.005	3	0.52 (0.38 to 0.72)	−0.88 (−1.52 to −0.23)	.008
ICs and TCs	6	0.79 (055 to 1.14)	−0.45 (−1.08 to 0.18)	.16	7	0.84 (0.70 to 1.02)	−0.45 (−1.13 to 0.22)	.18
TCs	13	1.22 (0.87 to 1.70)	0 [Reference]	NA	9	1.35 (0.93 to 1.95)	0 [Reference]	NA
Tumor locality								
HNSCC	4	1.27 (0.81 to 1.98)	0 [Reference]	NA	2	1.22 (0.75 to 1.96)	0 [Reference]	NA
Oral cavity	10	0.96 (0.48 to 1.93)	−0.15 (−1.04 to 0.74)	.16	7	1.02 (0.16 to 6.44)	0.27 (−0.28 to 0.82)	.56
Oropharynx and hypopharynx	8	0.70 (0.58 to 0.85)	−0.12 (−0.71 to 0.46)	.25	10	0.80 (0.68 to 0.95)	−0.09 (−0.74 to 0.55)	.42
Treatment modality								
Surgery	13	1.07 (0.74 to 1.55)	−0.49 (−1.17 to 0.20)	.74	9	0.97 (0.63 to 1.50)	0.25 (−1.11 to 0.60)	.34
RT, RCHT, or both	3	0.60 (0.33 to 1.12)	0 [Reference]	NA	7	0.95 (0.73 to 1.24)	0 [Reference]	NA
Surgery and RCHT	6	0.88 (0.54 to 1.44)	−0.38 (−1.04 to 0.27)	.68	3	0.80 (0.41 to 1.54)	−0.27 (−0.93 to 0.38)	.78

Abbreviations: aHR, adjusted hazard ratio; IC, immune cell; HNSCC, head and neck squamous cell carcinoma with no specified site; NA, not applicable; PD-L1, programmed cell death ligand 1; RT, radiotherapy; RCHT, radiochemotherapy; TC, tumor cell.

Primary outcome results were supported by secondary analysis results. More favorable specific survival was found exclusively in patients with high levels of PD-L1 expression on immune cells (pooled aHR, 0.52; 95% CI, 0.38 to 0.72). In addition, these patients had longer survival compared with those with PD-L1 expression on tumor cells, with a negative metaregression coefficient (−0.88; 95% CI, −1.52 to −0.23; *P* = .008).

### Interaction of Tumor Locality or Treatment Modality With Survival Associations

There was no interaction between cancer cite and the association of PD-L1 expression with OS or specific survival, although longer survival was observed in patients with oropharyngeal and hypopharyngeal cancers, with pooled aHRs of 0.70 (95% CI, 0.58-0.85) for OS and 0.80 (95% CI, 0.68-0.95) for specific survival. Nevertheless, outcomes of our metaregression showed no difference in survival among patients without a specified carcinoma site compared with those with oral cavity or oropharyngeal and hypopharyngeal carcinomas. In pooled aHRs and metaregression coefficients stratified by treatment modality (ie, radiotherapy or surgery followed by radiochemotherapy or surgery only), there was no interaction with the association between PD-L1 expression and OS or specific survival (Table 2).

### Quality of Evidence

While all studies exhibited low RoB using the Newcastle-Ottawa Quality Assessment Scale system (eTable 2 in Supplement 1), 7 studies^23,24,27,30,31,32,35^ showed moderate RoB that was associated with prognostic and confounding factors or analysis domains when using the QUIPS tool (eTable 3 in Supplement 1). The QUIPS-assessed RoB of studies focusing on the association between PD-L1 expression on immune cells and OS was low.

No publication bias was found among studies assessing PD-L1 expression on immune cells given that the studies met criteria of interstudy homogeneity, and no change in outcome was associated with small studies. Moreover, the estimated contribution of 2 missing studies did not change the primary outcome (imputed aHR, 0.43; 95% CI, 0.31-0.60). By contrast, studies assessing composite PD-L1 expression on both cell types had publication bias associated with different results of the DerSimonian-Laird and inverse variance models. In addition, the fixed-effects models suggested an association as documented by a pooled aHR of 0.76 (95% CI, 0.62-0.94). Nevertheless, no changes in outcomes associated with small studies or missing studies were found.

Likewise, no publication bias was noted in studies with PD-L1 measured exclusively on tumor cells. That is, there was no difference between fixed- and random-effects models and no change in outcome associated with small studies, and there were no missing unpublished studies.

## Discussion

This systematic review and meta-analysis found that PD-L1 expression levels were favorably associated with OS and specific survival when analyzing PD-L1 expressed only on infiltrating immune cells. The outcome of OS and specific survival estimated a reduction in risk of death by 61% and 48%, respectively. Composite PD-L1 expression on tumor and immune cells was not associated with improved survival.

There was no interaction between cancer site in patients with HNSCC and the association between PD-L1 expression and OS or specific survival given that metaregression coefficients did not demonstrate significantly shorter or prolonged survival in patients with oropharyngeal, hypopharyngeal, or oral cavity cancer compared with patients with no site specification. Our metaregression did not suggest whether there was in interaction between treatment modality and the association of PD-L1 and survival because survival stratified by patients undergoing surgery alone or with subsequent radiochemotherapy vs those with radiotherapy did not differ. Our outcomes were independent of tumor grade and stage and patient HPV, smoking, and alcohol consumption status given that the meta-analysis was conducted using only adjusted estimates. We can only speculate whether different immunohistochemical protocols of PD-L1 expression determination, including the cutoff value of positivity, may invert the outcome of our meta-analysis.

Previous meta-analyses in patients with nonmetastatic HNSCC did not show a prognostic potential of PD-L1 expression in association with OS.^36,37,38,39,40^ The reason for this may be the cell type specification of PD-L1 expression as documented in our meta-analysis.

Moreover, shortened survival was reported in a study^36^ assessing the association of low CD8+ tumor–infiltrating T lymphocytes and PD-L1 expressed on tumor cells with OS and was consistent with our subresult. Weaknesses of recently published meta-analyses may be that they were syntheses of low- to moderate-quality studies with insufficient or unavailable follow-up, missing survival data, or results obtained from univariate analyses.

### Limitations

Although our systematic review and meta-analysis suggested PD-L1 expression on immune cells as a novel prognostic biomarker, the strength of evidence was limited by the small number of studies assessing specific PD-L1 expression. Nevertheless, these studies exhibited interstudy homogeneity and no publication bias. Therefore, future studies may confirm the consistency of our findings as suggested by the pooled aHR of imputed missing studies.

## Conclusions

This systematic review and meta-analysis of 17 studies^19,20,21,22,23,24,25,26,27,28,29,30,31,32,33,34,35^ found that high PD-L1 expression levels on immune cells in the tumor immune microenvironment were associated with extended OS and specific survival in patients with localized and locoregionally advanced HNSCC. Further studies focused on this objective may be warranted to investigate whether high PD-L1 levels on immune cells may serve as a reliable and widely recognized prognostic marker.

## Notes

Supplement 1. **eTable 1.** Search Strategy **eTable 2.** Newcastle-Ottawa Scale Assessment of Quality of Included Studies **eTable 3.** Quality Assessment of Risk of Bias of Included Studies With Quality in Prognosis Studies Tool

Supplement 2.
Data Sharing Statement
