# Supplementary material for: Programmed Cell Death Ligand 1 Expression on Immune Cells and Survival in Patients With Nonmetastatic Head and Neck Cancer: A Systematic Review and Meta-analysis
Source: JAMA Netw Open. 2023 Mar 31;6(3):e236324. doi: 10.1001/jamanetworkopen.2023.6324 (PMC10066461; doi:10.1001/jamanetworkopen.2023.6324)
Supplement: Supplement 1. — eTable 1. Search Strategy eTable 2. Newcastle-Ottawa Scale Assessment of Quality of Included Studies eTable 3. Quality Assessment of Risk of Bias of Included Studies With Quality in Prognosis Studies Tool [file jamanetwopen-e236324-s001.pdf]

Supplemental Online Content

Blažek T, Petráš M, Knybel L, Cvek J, Soumarová R. Programmed cell death ligand 1 expression on immune cells and survival in patients with nonmetastatic head and neck cancer: a systematic review and meta-analysis. *JAMA Netw Open.* 2023;6(3):e236324. doi:10.1001/jamanetworkopen.2023.6324

eTable 1. Search Strategy

eTable 2. Newcastle-Ottawa Scale Assessment of Quality of Included Studies

eTable 3. Quality Assessment of Risk of Bias of Included Studies With Quality in Prognosis Studies Tool

This supplemental material has been provided by the authors to give readers additional information about their work.

© 2023 Blažek T et al. *JAMA Network Open.*

eTable 1. Search Strategy

**PubMed database**
(("Squamous Cell Carcinoma of Head and Neck"[Mesh] OR "Head and Neck Neoplasms"[Mesh] OR "Hypopharyngeal Neoplasms"[Mesh] OR "Otorhinolaryngologic Neoplasms"[Mesh] OR "Laryngeal Neoplasms"[Mesh] OR "Oropharyngeal Neoplasms"[Mesh] OR "Pharyngeal Neoplasms"[Mesh] OR "Gingival Neoplasms"[Mesh] OR "Palatal Neoplasms"[Mesh] OR "Parotid Neoplasms"[Mesh] OR "Mouth Neoplasms"[Mesh] OR "Tongue Neoplasms"[Mesh] OR "Tonsillar Neoplasms"[Mesh]) AND ("B7-H1 Antigen"[Mesh] OR "CD274 protein, human" [Supplementary Concept]) AND ("2010/01/01"[Date - Publication] : "2023/01/05"[Date - Publication]))
**MEDLINE STNext database**
L1 QUE 2010-2022/PY
L2 QUE "SQUAMOUS CELL CARCINOMA OF HEAD AND NECK"/CT OR "HEAD AND NECK NEOPLASMS"/CT OR "HYPOPHARYNGEAL NEOPLASMS"/CT OR "OROPHARYNGEAL NEOPLASMS"/CT OR "LARYNGEAL NEOPLASMS"/CT OR "MOUTH NEOPLASMS"/CT OR "TONGUE NEOPLASMS"/CT
L3 QUE HEAD OR NECK OR OTORHINOLARYNG* OR ORAL(W)CAVIT* OR OROPHARYNG* OR HYPOPHARYNG* OR LARYN* OR "ALVEOLAR RIDGE" OR TONGUE OR PALATE OR MOUTH OR TONSILLAR
L4 QUE *CANCER* OR *CARCINO* OR *NEOPLAS* OR *MALIGNAN* OR *TUMOR* OR *TUMOUR* OR MALIGNAN* OR ONCO* OR *LEUKEM* OR CHEMOTHERAP* OR CHEMO
L5 QUE "B7-H1 ANTIGEN"/CT
L6 QUE PD(W)"L1" OR DEATH(W)"L1" OR DEATH(W)LIGAND(W)1 OR B7(W)H1 OR B7(W)HOMOLOG(W)1 OR CD274
L7 861 SEA (L2 OR L3(3A)L4) AND (L5 OR L6/TI,AB) AND L1
**EMBASE STNext database**
L8 QUE "HEAD AND NECK CANCER"/CT OR "HEAD AND NECK TUMOR"/CT OR "HEAD AND NECK CANCER CELL LINE"/CT OR "BENIGN HEAD AND NECK TUMOR"/CT OR "MOUTH CANCER"/CT OR "MOUTH TUMOR"/CT OR "TONGUE CANCER"/CT OR "TONGUE TUMOR"/CT
L9 QUE "PD L1 PROTEIN"/CT OR "PD L1 GENE"/CT OR "B7 H1 ANTIBODY"/CT OR "B7 H1 GENE"/CT OR "B7 H1 RECEPTOR"/CT
L10 QUE "PROGRAMMED DEATH 1 LIGAND 1 INHIBITOR"/CT OR "PROGRAMMED DEATH LIGAND 1 ANTIBODY"/CT OR "PROGRAMMED DEATH LIGAND 1 INHIBITOR"/CT OR "PROGRAMMED DEATH RECEPTOR 1 ANTIBODY"/CT
L11 1204 SEA (L8 OR L3(3A)L4) AND (L9 OR L10 OR L6/TI,AB) AND L1
**PQSCITECH STNext database**

© 2023 Blažek T et al. *JAMA Network Open.*

L12	166 SEA L3(3A)L4 AND L6/TI,AB AND L1
**HCAPLUS STNext database**	
L13	QUE "HEAD AND NECK SQUAMOUS CELL CARCINOMA"/CT OR "OROPHARYNGEAL NEOPLASM"/CT OR "HEAD AND NECK NEOPLASM"/CT OR "HYPOPHARYNGEAL NEOPLASM"/CT OR "LARYNGEAL NEOPLASM"/CT OR "MOUTH NEOPLASM"/CT OR "TONGUE NEOPLASM"/CT
L14	QUE "PROGRAMMED DEATH-LIGAND PD-L1"/CT OR "PROGRAMMED DEATH-LIGAND 1 INHIBITORS"/CT OR "PROGRAMMED DEATH-LIGAND 1"/CT
L15	858 SEA ((L13 OR L3(3A)L4) AND (L14 OR L6/TI,AB) AND L1) NOT P/DT

© 2023 Blažek T et al. *JAMA Network Open.*

eTable 2. Newcastle-Ottawa Scale Assessment of Quality of Included Studies

Study	Selection				Comparability		Outcome		Total
	Representativeness of the exposed cohort	Selection of the non-exposed cohort	Ascertainment of exposure	Demonstration that outcome of interest was not present at start of study	Comparability of cohorts on the basis of the design or analysis controlled for confounders	Assessment of outcome	Was follow-up long enough for outcomes to occur	Adequacy of follow-up of cohorts	Score of stars
Adamski et al, 2021	*	*	*	*	* *	*	*	*	9
Fu et al, 2022	*	*	*	*	*	*	*	*	8
Fukushima et al, 2018	*	*	*	*	**	*	*	*	9
Hanna et al, 2018	*	*	*	*	**	-	*	*	8
Hong et al, 2016	*	*	*	*	**	*	*	*	9
Kim et al, 2016	*	*	*	*	**	*	*	*	9
Lin et al, 2015	*	*	*	*	*	*	*	*	8
Lyu et al, 2019	*	*	*	*	**	-	*	*	8
Pena-Cardelles et al, 2022	*	*	*	*	**	*	*	*	9
Sato et al, 2019	*	*	*	*	**	*	*	*	9
Schenider et al, 2018	*	*	*	*	*	*	*	*	8
Yang et al, 2018	*	*	*	*	**	*	*	*	9
Zhou et al, 2020	*	*	*	*	**	*	*	*	9
Balermpas et al, 2017	*	*	*	*	**	*	*	*	9
Lilja-Fischer et al, 2020	*	*	*	*	*	*	*	*	8
Sánchez-Canteli et al, 2020	*	*	*	*	*	*	*	*	8
Ngamphaiboon et al, 2019	*	*	*	*	**	*	*	*	9

* The studies were awarded stars in all categories with a maximum of 2 stars in category comparability of cohorts on the basis of the design or analysis. The total score ranged from 0 to 9 stars, and studies with a low risk of bias were awarded at least 7 stars.

© 2023 Blažek T et al. *JAMA Network Open.*

eTable 3. Quality Assessment of Risk of Bias of Included Studies With Quality in Prognosis Studies Tool

**Study**	**Study Participation**	**Study Attrition**	**Prognostic factor**	**Outcome**	**Study con-founding**	**Analysis and reporting**	**Total Risk of Bias**
Adamski et al, 2021	○	○	○	○	○	○	Low
Fu et al, 2022	○	○	○	○	◐	○	Moderate
Fukushima et al, 2018	○	○	○	○	○	○	Low
Hanna et al, 2018	○	○	○	○	○	◐	Moderate
Hong et al, 2016	○	○	○	○	○	○	Low
Kim et al, 2016	○	○	○	○	○	○	Low
Lin et al, 2015	○	○	◐	○	○	○	Moderate
Lyu et al, 2019	○	○	○	○	○	◐	Moderate
Peña-Cardelles et al, 2022	○	○	○	○	○	○	Low
Sato et al, 2019	○	○	○	○	○	○	Low
Schenider et al, 2018	○	○	○	○	◐	○	Moderate
Yang et al, 2018	○	○	○	○	○	○	Low
Zhou et al, 2020	○	○	○	○	○	○	Low
Balermpas et al, 2017	○	○	○	○	○	○	Low
Lilja-Fischer et al, 2020	○	○	○	○	◐	○	Moderate
Sánchez-Canteli et al, 2020	○	○	○	○	◐	○	Moderate
Ngamphaiboon et al, 2019	○	○	○	○	○	○	Low

The risk of bias for each domain was determined by counting the number of inadequate "no" rated items in each domain, with each domain containing 3–7 items.

○ – domain was rated as low risk, when there was no inadequate item

◐ – domain was rated as moderate risk, when there was at least one inadequate "no" rated item, with a maximum of 2–3, depending on the domain typ

© 2023 Blažek T et al. *JAMA Network Open.*
