# Supplementary material for: Programmed Cell Death Ligand 1 Expression on Immune Cells and Survival in Patients With Nonmetastatic Head and Neck Cancer: A Systematic Review and Meta-analysis
Source: JAMA Netw Open. 2023 Mar 31;6(3):e236324. doi: 10.1001/jamanetworkopen.2023.6324 (PMC10066461; doi:10.1001/jamanetworkopen.2023.6324)
Supplement: Supplement 2. — Data Sharing Statement [file jamanetwopen-e236324-s002.pdf]

Data Sharing Statement

Blažek. Programmed Cell Death Ligand 1 Expression on Immune Cells and Survival in Patients With Nonmetastatic Head and Neck Cancer: A Systematic Review and Meta-analysis. *JAMA Netw Open*. Published March 31, 2023. doi:10.1001/jamanetworkopen.2023.6324

Data
Data available: Yes
Data types: Deidentified participant data
How to access data: Data will be availabe on the request on e-mail address- - tomas.blazek@fno.cz
When available: With publication
Supporting Documents
Document types: None

Additional Information
Who can access the data: Data will be available to researchers whose proposed use of the data has been approved.
Types of analyses: Data from includede studies and statistical analyses will be available for any purpose.
Mechanisms of data availability: Data will be available after approval with signed data access agrreement.
